# Variety of Work in a Base Military Hospital

**Published:** 1915-11

**Authors:** Marion C. Pruitt

**Affiliations:** Atlanta, Ga.; Resident Surgical Officer Westminster Hospital, London, Eng.


### Journal-Record of Medicine

Successor to Atlanta Medical and Surgical Journal, Established 1855
and Southern Medical Record, Established 1870

**OWNED BY THE ATLANTA MEDICAL JOURNAL COMPANY**

PUBLISHED MONTHLY

*Official Organ Fulton County Medical Society, State Examing Board, Atlanta, Birmingham and Atlantic Railroad Surgeon's Association, Chattahoochee Valley Medical and Surgical Association, Etc.*

RICHARD R. DALY, M. D., Editor
BERNARD WOLFF, M. D., Supervising Editor
A. W. STIRLING, M. D., C. M., D. P. H.; J. S. HURT, B. Ph., M.D.
GEO. M. NILES, M. D., W. J. LOVE, M. D., (Ala.); Associate Editors
E. W. ALLEN, Business Manager

**COLLABORATORS**

W. F. WESTMORELAND, M. D., General Surgery
F. W. McRAE, M. D., Abdominal Surgery
ALLEN H. BUNCE, Medical Societies
E. B. BLOCK, M. D., Diseases of the Nervous System
MICHAEL HOKE, M. D., Orthopedic Surgery
CYRUS W. STRICKLER, M. D., Legal Medicine and Medical Legislation
E. C. DAVIS, A. B., M. D., Obstetrics
E. G. JONES, A. B., M. D., Gynecology
ALLEN H. BUNCE, M. D., Pathology and Bacteriology
R. T. DORSEY, JR., B. S., M. D., Medicine
L. M. GAINES, A. B., M. D., Internal Medicine
GEO. C. MIZELL, M. D., Diseases of the Stomach and Intestines
L. B. CLARKE, M. D., Pediatrics
EDGAR PAULLIN, M. D., Opsonic Medicine
THEODORE TOEPEL, M. D., Mechano Therapy
R. R. DALY, M. D., Diseases of the Eye, Ear, Nose and Throat
BERNARD WOLFF, M. D., Diseases of the Skin
E. G. BALLENGER, M. D., Diseases of the Genito-Urinary Organs

VOL. LXII. ATLANTA, GA., NOVEMBER, 1915. NO. 8

### VARIETY OF WORK IN A BASE MILITARY HOSPITAL.

MARION C. PRUITT, L. R. C. P., S., M. D., Atlanta, Ga.; Resident Surgical Officer Westminster Hospital, London, Eng.

My object of this report is to give to the medical profession an idea as to the variety of work that is done in a Base Military hospital.

As I have only been Resident Surgical Officer for three months and 50 per cent. of our work is civilian, the report is necessarily small but it gives a clear idea as to the kind of work done.

The cases are from 2 to 7 days and longer before they arrive at this hospital and come from France (90%) and Dardenalles (10 per cent.). Many are put on hospital ships the same day they receive the wound; others are treated (first aid) in first Base Hospitals and then sent on as soon as possible.

Condition of wounds: 98 per cent. were infected; chiefly mixed infection and frequently very extensive, thus: cellulitis of hand, arm and forearm from a S. W. of hand, or leg and thigh from a G. S. W. of foot. A S. W. (gunshot wound) includes bullets, shrapnel, bombs, or any kind of high explosives, as it is impossible to distinguish the difference unless you find the bullet or as the case may be; and then you may not be able to say definitely.

I have taken the different anatomical regions as a key, and classified my cases from this. This gives an idea as to the part of the body most exposed or wounded. It is to be noted that wounds of the vital structures are usually fatal and are not considered as wounded, but killed.

| | |
|---|---|
| Total No. of Surgical Cases | 314 |
| Total No. died, Surgical cases | 7 |
| Total of Medical Cases | 95 |
| Total of Medical Cases, died | 1 |
| Total No. of Medical and Surgical | 409 |
| Total No. of Medical and Surgical, died | 8 |
| Mortality (medical and surgical) | 1.99 per cent. |

SURGICAL CASES.

| Anatomical Region. | Diagnosis. | No. of Cases. | Died |
|---|---|---|---|
| Scalp | G. S. W. | 4 | ---- |
| Skull | G. S. W. | 5 | 1 |
| Eye | G. S. W. | 4 | ---- |
| Eye | G. S. W. | 4 | ---- |
| Eye | Astigmatism | 1 | ---- |
| Ear | Tobacco Amblyopia | 1 | ---- |
| Mandible | Middle ear disease | 5 | ---- |
| Mandible & ear & face | Fracture—hand grenade | 1 | ---- |
| Scalp and leg | G. S. W. | 1 | ---- |
| Head and Chest | Multiple G. S. W. | 1 | ---- |
| Head and Back | Multiple G. S. W. | 1 | ---- |

| | | | |
|---|---|---|---|
| Face and hands | Multiple G. S. W. | 1 | ---- |
| Face and shoulder | Multiple G. S. W. | 1 | ---- |
| | Multiple G. S. W. | 1 | ---- |
| Neck | Multiple Burns, High Explosive | 1 | ---- |
| Neck and spine | G. S. W | 8 | ---- |
| Neck and forearm | Multiple G. S. W. | 2 | ---- |
| Shoulder | Multiple G. S. W. | 1 | ---- |
| Shoulder | G. S. W. | 14 | ---- |
| Shoulder & arm | Dislocation | 1 | ---- |
| Shoulder, & arm & eye | Multiple G. S. W. | 1 | ---- |
| Shoulder & back | Multiple G. S. W. | 1 | ---- |
| Shoulder & gas-poison | Multiple G. S. W. | 1 | ---- |
| Shoulder & Buttock, (gas-gangrene) | Multiple G. S. W. | 1 | ---- |
| Arm | Multiple G. S. W. | 1 | 1 |
| Arm | G. S. W. | 9 | ---- |
| Both Arms | G. S. W. and fracture | 9 | 1 |
| Arm & forearm | G. S. W. and fracture | 1 | ---- |
| Arm & forearm frac. | Multiple G. S. W. | 2 | ---- |
| Arm & Breast | G. S. W. | 1 | ---- |
| Arm | Scald | 1 | ---- |
| Elbow | G. S. W. | 4 | ---- |
| Forearm | G. S. W. | 12 | ---- |
| Forearm | G. S. W. fracture | 3 | ---- |
| Hand, finger or both | G. S. W. | 24 | ---- |
| Hand & arm | Bruise | 2 | ---- |
| Back (muscles) | G. S. W. | 10 | ---- |
| Thoracic Cavity | G. S. W. | 9 | ---- |
| Back | Laceration | 4 | ---- |
| Thoracic Cav. & arm | Multiple G. S. W. | 2 | ---- |
| Back and arm | Multiple G. S. W. | 1 | ---- |
| Spine | G. S. W. | 1 | ---- |
| Abdominal wall | Hernia—Ing. | 4 | ---- |
| Abdominal Cavity | G. S. W. | 2 | ---- |
| Appendix | Appendicitis | 1 | ---- |
| Scrotum | Varicocele | 1 | ---- |
| Testicle | Orchitis Traumatis | 1 | ---- |
| Testicle | G. S. W. | 1 | ---- |
| Testicle, penis & thigh | Multiple G. S. W. | 1 | ---- |
| Rectum | Hemorrhoids | 4 | ---- |
| Perineum | G. S. W. | 1 | ---- |
| Pelvis | Fracture | 1 | ---- |
| Bladder | Pappilloma | 1 | 1 |
| Hip | G. S. W. | 3 | ---- |
| Buttock | G. S. W. | 5 | ---- |
| Thigh | G. S. W. (50 per cent. fractured) | 32 | ---- |
| Thigh & hand | Multiple G. S. W. | 3 | ---- |
| Thigh, chest & arm | Multiple G. S. W. | 1 | ---- |
| Thigh & leg | Multiple G. S. W. | 3 | ---- |
| Thigh & elbow | Multiple G. S. W. | 1 | ---- |
| Thigh, shoulder & arm | Multiple G. S. W. | 2 | 1 |
| Thigh & sacrum | Multiple G. S. W. | 1 | ---- |
| Inguinal | Glands | 1 | ---- |
| Thigh | Traumatic Aneurism (femoral) | 1 | ---- |

| | | | |
|---|---|---|---|
| **Knee** | G. S. W. | 11 | ---- |
| **Knee** | Injury | 3 | ---- |
| **Knee** | Dislocated semiluma cartilege | 2 | ---- |
| **Leg** | G. S. W. | 12 | ---- |
| **Leg** | G. S. W. and fracture | 16 | ---- |
| **Leg & feet** | Multiple G. S. W. & fracture | 1 | 1 |
| **Feet** | G. S. W. | 13 | 1 |
| **Feet** | Sore | 4 | ---- |
| **Feet** | Crushed | 3 | ---- |
| **Feet & shoulder** | G. S. W. | 3 | ---- |
| **Ankle** | Potts fracture | 1 | ---- |
| **Ankle** | Sprained | 1 | ---- |
| **Feet** | Trench foot | 18 | ---- |
| | Total | 314 | 7 |

MEDICAL CASES.

| **Diagnosis.** | **No. of Cases.** | **Died** |
|---|---|---|
| Pericarditis | 1 | ---- |
| Rheumatism | 29 | ---- |
| Heart Valve | 6 | ---- |
| Bronchitis | 9 | ---- |
| Dysentery | 14 | ---- |
| Laryngitis | 2 | ---- |
| Typhoid | 2 | ---- |
| Tuberculosis | 2 | 1 |
| Gas-poison | 4 | ---- |
| Shell-shock | 4 | ---- |
| Epilepsy | 1 | ---- |
| Cholecystitis | 1 | ---- |
| Tonsilitis and Dyspepsia | 2 | ---- |
| Nephritis | 3 | ---- |
| Scabes | 3 | ---- |
| Pleurisy | 2 | ---- |
| Para-typhoid | 2 | ---- |
| Debility | 2 | ---- |
| Pyrexia | 2 | ---- |
| Bronchitis and Trench foot | 2 | ---- |
| Anaemia Sec. | 1 | ---- |
| Pneumonia | 1 | ---- |
| Total | 95 | 1 |

